# Translational Repression in Malaria Sporozoites

**DOI:** 10.15698/mic2016.05.502

**Published:** 2016-04-05

**Authors:** Oliver Turque, Tiffany Tsao, Thomas Li, Min Zhang

**Affiliations:** 1 HIV and Malaria Vaccine Program, Aaron Diamond AIDS Research Center, Affiliate of The Rockefeller University, New York, NY, USA.; 2 Department of Pathology, New York University School of Medicine, New York, NY, USA.

**Keywords:** Plasmodium, sporozoites, latency, eIF2α, UIS1, UIS2, translational repression

## Abstract

Malaria is a mosquito-borne infectious disease of humans and other animals. It is caused by the parasitic protozoan, *Plasmodium*. Sporozoites, the infectious form of malaria parasites, are quiescent when they remain in the salivary glands of the *Anopheles* mosquito until transmission into a mammalian host. Metamorphosis of the dormant sporozoite to its active form in the liver stage requires transcriptional and translational regulations. Here, we summarize recent advances in the translational repression of gene expression in the malaria sporozoite. In sporozoites, many mRNAs that are required for liver stage development are translationally repressed. Phosphorylation of eukaryotic Initiation Factor 2α (eIF2α) leads to a global translational repression in sporozoites. The eIF2α kinase, known as Upregulated in Infectious Sporozoite 1 (UIS1), is dominant in the sporozoite. The eIF2α phosphatase, UIS2, is translationally repressed by the Pumilio protein Puf2. This translational repression is alleviated when sporozoites are delivered into the mammalian host.

*Plasmodium* sporozoites are quiescent for several weeks in mosquito salivary glands while maintaining their infectivity. In stark contrast to the parasite’s continuous transcriptional changes in the liver (pre-erythrocytic) and blood (erythrocytic) stages in their vertebrate hosts, the sporozoite’s transcriptional program remains unchanged during the long period of time in mosquito salivary glands. The transcriptional and translational profiles of sporozoites and pre-erythrocytic stage parasites display significant lags in protein abundance relative to mRNA abundance, due to a global translational repression caused by the phosphorylation of eIF2α in salivary gland sporozoites.

Eukaryotic Initiation Factor 2 (eIF2), composed of α, β, γ subunits, recruits Met-tRNA and GTP to form a ternary complex that binds to ribosome, which initiates protein translation. GTP is then hydrolyzed, and the ribosome releases eIF2-GDP as an inactive binary complex. EIF2B, a guanine nucleotide exchange factor, subsequently converts eIF2-GDP to eIF2-GTP, which is required for another round of translational initiation. Phosphorylation of eIF2α blocks the recycling of eIF2-GDP to its translationally active eIF2-GTP form, thereby inhibiting global protein synthesis. The stalled mRNAs are then assembled as stress granules (messenger ribonucleoprotein complexes) in *Plasmodium* sporozoites. Activating Transcription Factor 4 (ATF4), a transcriptional activator of genes involved in the integrated stress response, is preferentially translated when eIF2α is phosphorylated in mammalian cells (Figure 1A). In *Plasmodium*, it is unknown if phosphorylation of eIF2α facilitates the preferential translation of select transcripts while repressing general translation initiation.

**Figure 1:**
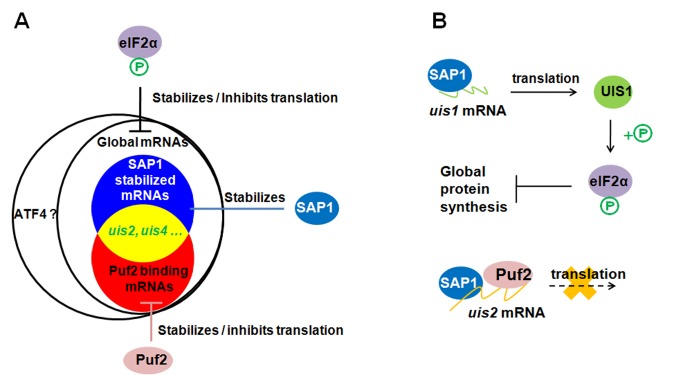
FIGURE 1: Model illustrating translational repression in malaria sporozoites. **(A)** Translational repression by eIF2α phosphorylation and Puf2. Puf2 stabilizes and inhibits translation of a group of mRNAs by binding to their 3' untranslated region. SAP1 stabilizes 38 *uis* mRNAs and non-*uis* mRNAs. EIF2α phosphorylation inhibits global protein synthesis.The stalled mRNAs are assembled into RNA granules. **(B)** Regulation of eIF2α phosphorylation. Phosphorylation of eIF2α is controlled by the kinase UIS1 and the phosphatase UIS2. *uis1* and *uis2* transcripts are stabilized by SAP1, and the translation of *uis2* is repressed by Puf2. UIS1 is dominant, and causes dormancy in *Plasmodium* sporozoites.

*Plasmodium* eIF2α kinase UIS1 (also termed eIK1) phosphorylates eIF2α in salivary gland sporozoites. *Uis* genes are a subset of thirty genes that are transcriptionally upregulated when sporozoites differentiate from the mosquito midgut to salivary glands. The eIF2α phosphatase is UIS2 in *Plasmodium* salivary glands sporozoites, where the *uis1* and *uis2* mRNAs are most abundant among the *uis* transcripts. *Plasmodium* Pumilio protein Puf2 contains a sequence-specific RNA binding domain and serves as a translational repressor of specific mRNAs by binding to their 3'-untranslated region. Puf2 inhibits translation of *uis2*, consequently rendering UIS1 dominant. This leads to a prevalence of phosphorylated eIF2α and global protein repression in sporozoites (Figure 1B).

Many liver stage messages, such as *uis2* and *uis4*, bind to Puf2 and are translationally repressed and stabilized in sporozoites (Figure 1). The transcriptome and morphology of *puf2*(-) sporozoites in *P. berghei* and *P. yoelii* rodent malaria, transform into early liver stages. The transcript of *uis1* is degraded in the *puf2*(-) sporozoites. The phenotype of the eIF2α kinase *uis1*(-) is similar to that of *puf2*(-). The eIF2α phosphatase *uis2*(-) sporozoites do not transform into liver stages but maintain their crescent sporozoite shapes after delivery to host cells. The phenotype of *uis2*(-) is adverse to that of *uis1*(-) and *puf2*(-).

Sporozoite Asparagine-rich Protein 1 (SAP-1) is essential for the stability of a group of thirty-eight mRNA transcripts in *P. yoelli* salivary gland sporozoites. The transcripts of many *uis* genes are quickly degraded at their 3’-ends, including *uis1* and *uis2*. Additionally, in the *sap1*(-) sporozoite, some transcripts, including *uis4*, were shown to be quickly degraded at their 5’-ends. Thus, in the absence of SAP-1, genes that are essential to liver-stage development of the infectious sporozoite follow the pattern in which the 3’ and 5’ ends of the mRNA molecule are sequentially degraded.

Since UIS2 is essential for liver-stage development of sporozoites, we explored its function. In mammalian cells, Protein Phosphatase 1 (PP1) with its cofactor GADD34 or CReP mediates the dephosphorylation of eIF2α-P. However, GADD34 and CReP are absent in *Plasmodium*. Our data show that *Plasmodium* PP1 does not dephosphorylate *Plasmodium* eIF2α-P in sporozoites. Rather, UIS2 is the eIF2α-P phosphatase that directly binds to its substrate eIF2α-P. Our demonstration that UIS2 is a novel eIF2α-P phosphatase reveals another mechanism to control eIF2α dephosphorylation. Our findings also suggest that additional mechanisms have evolved to control eIF2α dephosphorylation in organisms that do not contain recognizable homologs of GADD34 or CReP.

UIS2 contains a conserved RVxF motif within its phosphatase domain (Figure 2). This motif is a putative PP1-binding motif. Although PP1 is not an eIF2α phosphatase in sporozoites, we do not exclude the possibility that *Plasmodium* PP1 might be an eIF2α phosphatase in other stages of the malaria life cycle. An interesting hypothesis requiring further investigation is that *Plasmodium* PP1 is recruited by UIS2. The redundant eIF2α phosphatase may result in rapid dephosphorylation of eIF2α during the sporozoite’s transition from its latent form to its active form. 

**Figure 2:**
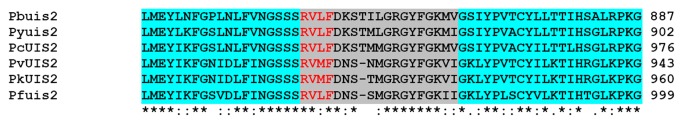
FIGURE 2: The RVxF motif in UIS2 phosphatase domain. *P. berghei* UIS2 (1321 amino acids) contains a phosphatase domain (roughly residues 535-1054). The amino acids around RVxF motif in UIS proteins from *P. berghei*, *P. yoelii*, *P. chabaudi*, *P. vivax*, *P. knowlesi*, and *P. falciparum* are aligned. The cyan area shows the conserved phosphatase domain and the gray region shows the insertion in UIS2 relative to 1UTE (the best matching phosphatase in Protein Data Bank).

*Plasmodium* sporozoites remain latent in mosquito salivary glands until transmission to a host. The translational repression of liver stage messages such as *uis2*, *uis3*, and *uis4* in sporozoites is correlated with the observation that these genes are not essential for the parasite’s sporozoite stage development. Liver stage messengers start to translate by the alleviation of translational repression. The initiation factor, eIF2α, is dephosphorylated in pre-erythrocytic stages where UIS2 is dominant. These parasites utilize continuous transcriptional changes for their pre-erythrocytic and erythrocytic stage development.

*P. vivax* infects humans and undergoes an especially dangerous type of latent morphology in the liver. Hypnozoites remain dormant in the liver of their host until they eventually cause a relapse of malaria. Though the transcriptional and translational profiles of hypnozoites are unknown, we speculate that translation is similarly repressed in hypnozoites, and that their latency is associated with eIF2α phosphorylation. One possibility is that UIS2 remains translationally repressed in hypnozoites until a signal (currently unknown) reactivates them.

